# Lentiviral and targeted cellular barcoding reveals ongoing clonal dynamics of cell lines *in vitro* and *in vivo*

**DOI:** 10.1186/gb-2014-15-5-r75

**Published:** 2014-05-30

**Authors:** Shaina N Porter, Lee C Baker, David Mittelman, Matthew H Porteus

**Affiliations:** 1 Department of Pediatrics, University of Texas Southwestern Medical Center, Dallas, TX 75390, USA; 2 Department of Pediatrics, Stanford Medical Center, Stanford, CA 94305, USA; 3 Virginia Bioinformatics Institute, Virginia Tech, Blacksburg, VA 24061, USA; 4 Department of Biological Sciences, Virginia Tech, Blacksburg, VA 24061, USA

## Abstract

**Background:**

Cell lines are often regarded as clonal, even though this simplifies what is known about mutagenesis, transformation and other processes that destabilize them over time. Monitoring these clonal dynamics is important for multiple areas of biomedical research, including stem cell and cancer biology. Tracking the contributions of individual cells to large populations, however, has been constrained by limitations in sensitivity and complexity.

**Results:**

We utilize cellular barcoding methods to simultaneously track the clonal contributions of tens of thousands of cells. We demonstrate that even with optimal culturing conditions, common cell lines including HeLa, K562 and HEK-293 T exhibit ongoing clonal dynamics. Starting a population with a single clone diminishes but does not eradicate this phenomenon. Next, we compare lentiviral and zinc-finger nuclease barcode insertion approaches, finding that the zinc-finger nuclease protocol surprisingly results in reduced clonal diversity. We also document the expected reduction in clonal complexity when cells are challenged with genotoxic stress. Finally, we demonstrate that xenografts maintain clonal diversity to a greater extent than *in vitro* culturing of the human non-small-cell lung cancer cell line HCC827.

**Conclusions:**

We demonstrate the feasibility of tracking and quantifying the clonal dynamics of entire cell populations within multiple cultured cell lines. Our results suggest that cell heterogeneity should be considered in the design and interpretation of *in vitro* culture experiments. Aside from clonal cell lines, we propose that cellular barcoding could prove valuable in modeling the clonal behavior of heterogeneous cell populations over time, including tumor populations treated with chemotherapeutic agents.

## Background

Even under ideal growth conditions, cultured cells exhibit genetic heterogeneity. It is therefore valuable, although technically challenging, to track the behavior and interplay of clones within a cellular population. Furthermore, clonal dynamics play important roles in cancer and stem cell biology. We therefore aimed to develop a sensitive and quantitative method for tracking the clonal dynamics within populations of cells with minimal disruption to both individual cells and the population as a whole.

Early techniques, able to track one or a few clones, relied upon gross chromosomal markers [1,2], heterozygous alleles [3,4], or a rainbow of fluorescent markers [5]. More recent methods have utilized viral integration to confer specific and theoretically unique heritable marks on a cell [6-9]. While these techniques greatly increase the number of clones that can be detected, the method is plagued by limitations in sensitivity and an inability to accurately measure the size of each clone, despite advances in detection [10-12]. To overcome these limitations, we decided to label cells with unique DNA barcodes, which can be recovered and sequenced to reveal the temporal and quantitative behavior of entire cell populations and also individual member clones.

The ability to track a limited subset of a cellular population with DNA barcodes has previously been demonstrated by several groups [13-17]. Here, we demonstrate the feasibility of monitoring entire cell populations using a barcode system that scales to many thousands or even a million individual clones. We also outline a novel non-viral barcoding method that targets barcodes to a single genomic locus through zinc-finger nuclease (ZFN)-induced homologous recombination and therefore avoids unpredictable viral insertional mutagenesis. With this more precise and scalable approach we are able to define the dynamics of an entire cell population rather than tracing the fates of only a few representative clones.

First, we validate the performance of our barcode method by tracking the *in vitro* dynamics of several common cell lines. We find that despite years in culture, common cell lines exhibit ongoing clonal instability. Next, we compare the clonal dynamics of cell populations barcoded by random insertion of a lentiviral vector versus targeted integration at a single genomic locus through homologous recombination and find that the nuclease-mediated insertion of the barcode sequence process itself results in dramatic changes in clonal representation. Finally, we measure the contributions of clones in primary xenograft tumors. By comparing the dynamics of the same population of clones *in vitro* and *in vivo*, we were able to show that the selective pressure that restricts clonal diversity is greater in culture than in a mouse xenograft. These findings add to our knowledge of *in vitro* and *in vivo* cellular behavior, and have important implications for the design and interpretation of experiments utilizing cultured cells.

## Results

### Library construction

**Figure 1:**
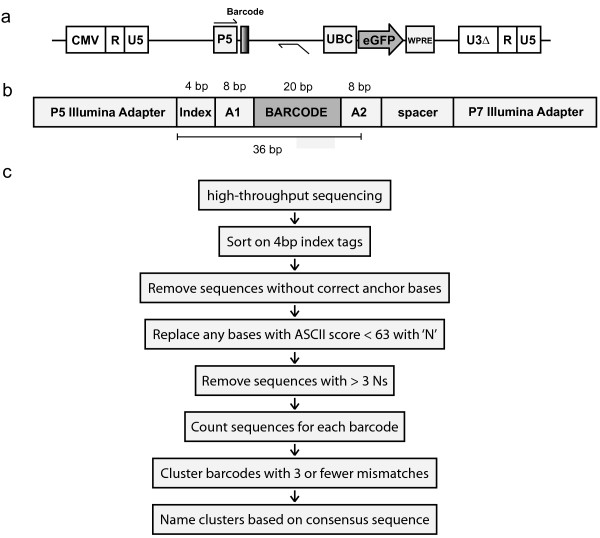
**Barcode lentiviral vector, sequencing and analysis workflow. (a)** The 20 bp DNA barcode was cloned into the non-coding region of a SIN (self-inactivating) lentiviral vector upstream of a UBC-eGFP cassette. The P5 Illumina sequencing adapter sequence was integrated next to the barcode, and the P7 adapter was added during the PCR amplification step (primer positions shown). **(b)** This PCR results in a 250 bp fragment that includes a 4 bp indexing tag to allow pooling of multiple samples into a single lane of a flow cell, in addition to the 20 bp random barcode sequence, and flanked on either side by eight 'anchor' bases, which act as markers to identify true barcode sequences within the sequencing data. Finally, the fragments contain a spacer of approximately 90 bp and the second (P7) Illumina adapter for sequencing. Integrating the adapter into the barcode vector allows for single-end 36 bp (short) sequencing reads in which the barcode end is always sequenced. **(c)** Data analysis workflow.

We genetically marked individual cells through transduction with a pool of lentivirus containing a library of unique 20 bp nucleotide sequences (termed barcodes). PCR amplification and high-throughput sequencing enable the resolution and quantification of individual barcodes within the population, thereby measuring both the absolute and relative abundance of every marked clone. We created barcodes by synthesizing a pool of oligonucleotides composed of 20 randomized bases flanked by defined static 'anchor' sequences. These anchor sequences allow us to identify and filter out contaminating sequence reads that do not contain barcodes. Double-stranded barcodes were cloned into the non-coding region of a self-inactivating lentiviral vector upstream of the enhanced green fluorescent protein (eGFP) transgene expressed from a ubiquitin C (UBC) promoter. The lentiviral vector was designed to include the Illumina P5 adapter sequence 8 bp upstream of the barcode sequence, facilitating amplification and sample preparation of the barcode sequences in a single PCR step, while positioning the barcode, allowing for the use of single-end 36 bp Illumina sequencing reads, and thus maximizing the barcode-to-cost ratio (Figure 1a). During PCR amplification of the barcodes with primers that contain both Illumina adapter sequences, 4 bp indexing tags are added to allow for pooling of multiple samples per flow cell lane. The resultant 250 bp fragment (Figure 1b) contains the indexing tag, 8 bp of anchor sequence, and the 20 bp barcode, flanked by the adapters.

### Library validation and data analysis pipeline

To determine the complexity and distribution of the barcode library, as well as to determine the extent of error and bias introduced by sample preparation and sequencing, we independently PCR-amplified the plasmid barcode library for sequencing four separate times, and sequenced each amplified sample on an independent flow cell lane at a coverage of 400-fold.

**Figure 2:**
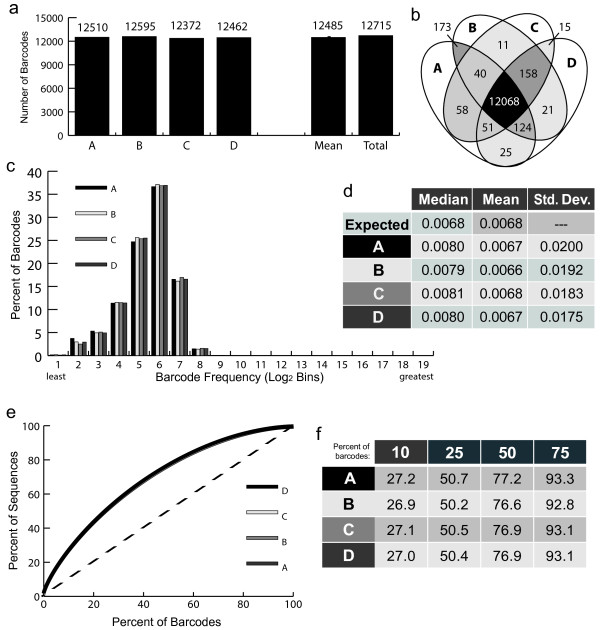
**Barcode plasmid library analysis.** Results from four separate PCR amplification and sequencing runs of the plasmid barcode library (A to D). **(a)** The number of barcodes found in each replicate after analysis and trimming. 'Mean' is the average number of barcodes for the four replicates; 'Total' is the number of unique barcodes found within the four samples combined. **(b)** Venn diagram demonstrating the amount of overlap of barcodes among the four replicates. Darker shading indicates larger numbers of barcodes. **(c)** Barcodes were counted and grouped in Log 2 bins based on percentage (frequency) within the population, from least to greatest. The percentage of the barcodes in each bin is shown. **(d)** The predicted (Expected) and experimentally determined median and mean barcode frequencies are shown as percentages, as well as the standard deviation from the mean. **(e)** The percentage of barcodes, ranked from most to least frequent plotted by what percentage of the total sequences they made up. Dashed line represents perfectly equal representation of barcodes. **(f)** The percentage of sequences made up by the top indicated percentages of the barcodes for each sample.

All computational methods for reading out the barcodes from raw Illumina FASTQ data are open source and available via Github at [18]. Briefly, we minimized misidentification of barcodes by replacing lower quality bases (those with a phred base quality of less than 30) with an ‘N’ to indicate uncertainty for that base (Figure 1c). Reads with more than 3 uncertain bases, with mismatches at any of the 12 anchor bases, or without a proper indexing tag were excluded from analysis. The remaining reads were trimmed to only include the 20 bp barcode sequence and then clustered according to the following rules: barcodes that contained 3 or fewer mismatches and 3 or fewer Ns were consolidated into a single cluster. Thus, the minimum number of base matches for two barcodes to be clustered as identical is 14 (20 possible - (3 mismatches) - (3 Ns)). The probability that any two barcodes in our barcode library with a complexity of approximately 12,500 matching at 14 out of 20 bp is low (0.00887). The size of the clones was determined by counting the number of reads in each cluster. We performed a doping experiment to measure lowest detectable barcode frequency and found that barcodes representing 0.0002% of the population were always detectable with our sequencing parameters, while less frequent barcodes were not always detected. This finding led us to implement a threshold for the detection of barcodes at 0.0002%.We applied our algorithm to the four plasmid library sequencing replicates (labeled A to D) and found that the number of barcodes in each sample was highly similar, with a mean of 12,485 barcodes and standard deviation of 93 barcodes (Figure 2a), while the total number of unique barcodes found in all four replicates was 12,715. In addition to the sequences trimmed by the analysis program, sequences were eliminated as noise if they did not appear in at least two of the four replicates. Less than 0.5% of barcodes were removed due to this restriction, and all appeared at very low frequency, suggesting that they resulted from sequence error rather than true, novel clones. The overall complexity of the barcode library that we use throughout this work is greater than 12,000. Figure 2b demonstrates the large degree of overlap among the barcodes found in each of the sequencing replicates, with 12,068 barcodes shared among all four replicates.

The distribution of barcodes between the four replicates was nearly identical as well. Sequences were counted, and then the frequency of each barcode was calculated as a percentage of the whole. The mean percent frequency of each replicate was very similar to the expected for a library this size (Figure 2d). The median barcode frequencies of the four replicates were also very similar to one another, spanning 0.0066% to 0.0068% with a low standard deviation (expected median frequency in an unskewed population = 0.0068%) (Figure 2d). By comparing the frequencies of each barcode in each of the sample replicates, we were able to determine R^2^ values, which ranged from 0.989 to 0.996 (Additional file 1). From this, we were able to conclude that our method of PCR amplification, sequencing and analysis is highly reproducible and does not introduce significant error or bias.Our measure and quantification of bias within the replicate barcode library sequences are shown in Figure 2c-f. Figure 2c shows a histogram of barcode frequency distribution in this library across all four sequencing replicates. A completely normal distribution would result in a bell shaped curve. Figure 2e plots the percentage of barcodes against the percentage of sequences and an unbiased distribution would result in a 45-degree line (dotted line). In both of these figures the slight skewing of the original plasmid library is demonstrated by the deviation from a bell shaped curve in 2c and the deviation from the 45-degree line in 2e. We quantify the bias in Figure 2f by plotting the percentage of sequences that were accounted for by 10, 25, 50, and 75% of the most abundant barcodes. In the original plasmid library the top 10, 25, 50, and 75% most abundant barcodes account for approximately 27, 50, 77, and 93% of the sequences, respectively, thus providing a quantitative metric of bias in barcode representation. This slight skewing in the plasmid barcode library is most likely the result of its amplification through overnight growth in bacteria as part of its preparation.

### Cellular barcode libraries and passaging experiments

**Figure 3:**
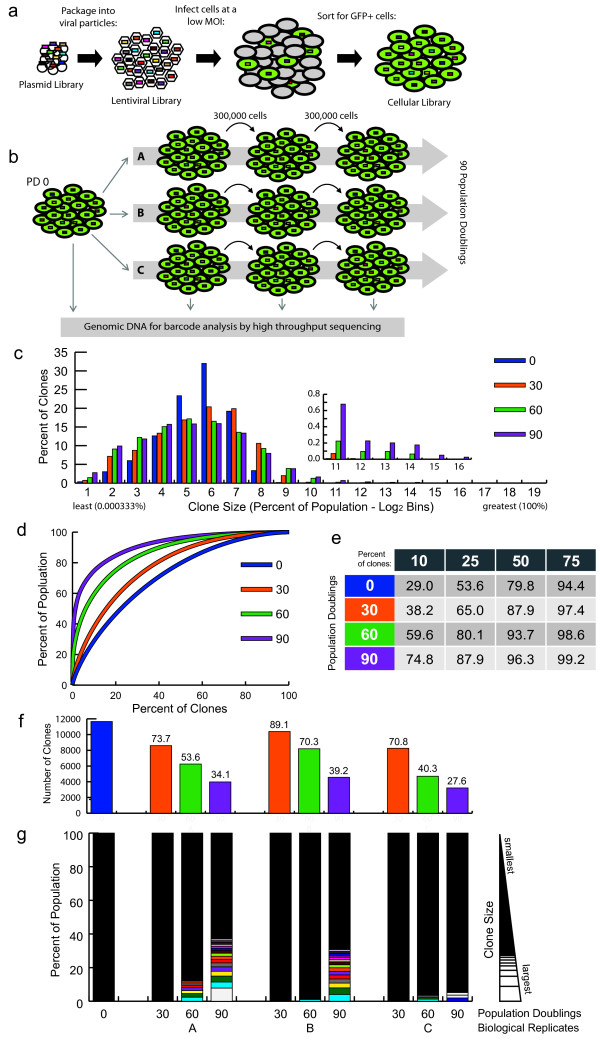
**K562 cellular barcode libraries. (a)** Workflow from plasmid barcode library to cellular barcode library. Unique barcodes are represented as different colored rectangles; barcoded cells also express eGFP. **(b)** Experimental design of passaging experiments. **(c)** Clones were counted and binned in Log 2 bins based on percentage (frequency) within the population, from least to greatest. The percentage of the clones in each bin is shown. Inset shows magnification of larger bins. K562 biological replicate A is shown (others are shown in Additional file 2). **(d)** The percentage of clones, ranked from most to least frequent, plotted by what percentage of the population they made up. **(e)** The percentage of the population made up by the top indicated percentages of clones for each sample. **(f)** The number of clones found in each sample. **(g)** Rank order barcodes by percentage of sequences for each sample; greatest to least. Any clones ≥1% are delimited by white sections within the column, while the remaining population of clones smaller than 1% are represented by the black area in each column. The same clone occurring as a major clone in more than one sample is identified by color.

For all cellular barcode libraries, cells were infected with lentivirus produced from the plasmid barcode library at a low multiplicity of infection (MOI; 0.05 to 0.1) to minimize the number of cells marked by multiple barcodes [19]. Four days after transduction, cells were sorted for GFP expression to enrich the population for barcode marked cells (Figure 3a). This population was expanded for several days and then 3 × 10^5^ cells (representing approximately 24 times the complexity of the barcode library) were taken to start each of three parallel cultures, known as biological replicates A, B, and C. Additionally, 3 × 10^5^ cells were harvested at this time point to determine the barcode distribution at the experimental start, termed 'population doubling 0' (PD 0). Every three days, cultured cells were counted and analyzed for GFP expression, mixed well, and passaged to fresh culture dishes (Figure 3b) maintaining a minimum of 3 × 10^5^ cells in log phase growth. In addition to PD 0, genomic DNA was harvested from a minimum of 10^6^ cells harvested when each population reached 30, 60, and 90 population doublings. The genomic DNA of 3 × 10^5^ cells from each time point was used as the template from which barcodes were PCR amplified for sequencing.

### K562 cellular barcode library passaging and results

For our first cellular barcode library and passaging experiments, we chose K562 cells, a common human leukemia cell line established in 1970 from a patient with chronic myelogenous leukemia [20]. We found that in all three biological replicates, the number of barcodes detected in each population decreased over time and the clonal distribution within each population became more biased over time as the tails become larger than would be expected from a normal distribution (Figure 3c-g, Additional file 2). Each of the replicates also contained clones that came to constitute greater than 1% of the total population ('major clones'), but all clones constituted less than 10% of their respective population at PD 90. At each time point, the clones identified were categorized as rare (less than 0.0007% of the population), abundant (greater than 0.5% of the population), or average (all others) based on their individual contribution to the total number of cells in culture (Additional file 3).

In order to determine whether the clonal dynamics within the three populations were due to pre-existing cell-intrinsic factors, or if the populations underwent clonal selection after the split, we compared the identities of the major clones in each replicate. One clone (Figure 3g, yellow) was found in all three populations as a major clone, suggesting that factors intrinsic to this cell at the time it was marked caused its progeny to have a growth advantage over its neighbors. However, most of the other major clones within each replicate were unique to that population, suggesting that each clone’s growth advantage was gained after the clone was marked and the biological replicates had been separated, indicating ongoing clonal variation followed by selection during the course of the experiment. As the population doubling increased, the most abundant clones contributed to a larger and larger portion of the total population (Figure 3d,e). For example, at PD 0 the 10% most abundant clones accounted for 29% of the total cells in the culture, but by PD 90 the top 10% now accounted for almost 75% of the total cells in the population (Figure 3e). Importantly, the 10% most abundant clones at PD 90 were not the same as the top 10% at PD 0. Furthermore, the dominant clones identified at PD 90 were derived from clones in all three percentage contribution categories (rare, average and/or abundant) at PD 0 in all three biological replicate populations (Additional file 4). The distribution of clones widened, with greater percentages of clones showing up in the highest and lowest bins, indicating an increasing trend in high and low frequency clones (Figure 3c). Thus, these experiments demonstrate that K562 cells continue to display rapid clonal dynamics even under optimal culturing and passaging conditions.

### K562 clonal cellular barcode library passaging and results

Since we observed ongoing clonal dynamics in our polyclonal K562 population, we hypothesized that this marked population of cells had developed significant heterogeneity over time from ongoing genetic and epigenetic changes that affected clonal fitness and dynamics. To test this hypothesis, we created a K562 line derived from a single cell, and repeated the barcoding experiment (as with the original K562 population). We found that although the rate of clone loss and diversification was slower, it still occurred (Additional files 5 and 6). There appears to be more overlap among the largest clones of the three biological replicates than seen with the polyclonal K562 cellular library, as well as a number of clones unique to each biological replicate, indicating ongoing clonal evolution). The slower but persistent changes observed in the population derived from a single cell are highlighted by the difference in percentage contribution of the top 10% most abundant clones identified. In the clonal K562 experiment, the top 10% of clones identified accounted for 32% of the population at PD 0 and 38% of the population at PD 90. This increase is dramatically less than that observed in the polyclonal K562 experiment wherein the top 10% most abundant barcodes accounted for 29% of the total sequences at PD 0 and 75% of the total sequences at PD 90 (compare Figure 3e with Additional file 5c).

### Targeted barcode library in K562 cells

**Figure 4:**
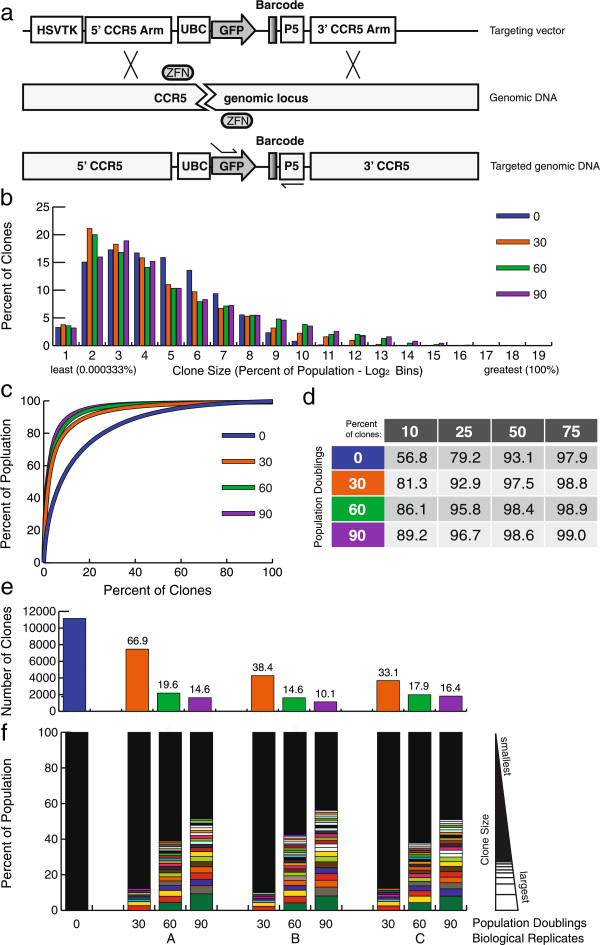
**Targeted barcode libraries in K562 cells. (a)** Schema for targeting barcodes to the *CCR5* locus. Targeting vector (repair template; top) includes a UBC-driven GFP gene upstream of a 20 bp barcode, and the P5 Illumina adapter sequence in reverse between CCR5 arms of homology. HSV-TK (herpes simplex virus thymidine kinase) is included outside of the arms of homology to allow drug selection against clones with off-target integration of the vector. Middle: the site of the ZFN-induced double strand DNA break. Bottom: the correctly targeted locus after homologous recombination with the targeting vector. **(b)** Clones were counted and binned in Log 2 bins based on percentage (frequency) within the population, from least to greatest. The percentage of the clones in each bin is shown. Inset shows magnification of larger bins. K562 biological replicate A of the CCR5-targeted barcode experiment is shown (others are shown in Additional file 7). **(c)** The percentage of clones, ranked from most to least frequent, plotted by what percentage of the population they made up. **(d)** The percentage of the population made up by the top indicated percentages of the clones in each sample. **(e)** The number of clones found in each sample. **(f)** Rank order clones by percentage of the population for each sample; greatest to least. Any clones ≥1% are delimited by white sections, the remaining population of clones smaller than 1% are represented by black in each column. The same clone occurring as a major clone in more than one sample is indicated with color.

While we utilized a lentiviral vector with self-inactivating long terminal repeats, the possibility remains that the insertion of our barcodes into the genomic DNA of a cell could result in genetic alterations that affect the behavior of individual clones [21,22]. In order to avoid insertional mutagenesis, we targeted gene integration to direct a second barcode library, with a similar complexity as the first (>12,000 barcodes), to a single genomic locus in K562 cells using homologous recombination and ZFNs (Figure 4a). In this manner, barcodes are inserted into the same genomic location within individual cells and thus variability caused by semi-random genomic insertion is eliminated. The genomic site we chose was the *CCR5* locus because it is considered a 'safe harbor' locus [23], meaning that disruption should not alter cellular phenotype. Furthermore, many reagents are available to effectively target this site [24-26]. While we, and others, have observed that the ZFNs targeting *CCR5* have some cellular toxicity, the effect on overall clonal dynamics was unknown and might be expected to be minimal [25]. We performed the targeting experiment at nuclease concentrations shown to favor single allele targeting to minimize double-marking cells [24]. After two pulses of ganciclovir to select against cells with off-target insertion of barcodes, GFP levels remained stable, suggesting that the majority of cells with off-target integrations had been eliminated. We used the same passaging strategy with these cells, except that we increased the number of cells maintained at each passage to 2 million cells (approximately 160-fold coverage) in larger volumes of media to maintain log-phase growth. Despite this increase, we saw rapid clonal loss and population skewing over the course of the experiment (Figure 4b-f, Additional file 7). In contrast to the three replicates using the lentiviral insertion of the barcode in which each replicate had its own unique signature of abundant clones, at each time point the three replicates with targeted integration of the barcode were nearly identical with respect to the size and identity of major clones. This indicates to us that the transient expression of the CCR5 ZFNs to initially target the barcode to the same genetic locus, the prerequisite capacity for efficient targeted integration by homologous recombination in these cells strongly influenced the clonal dynamics of the population before it was split, leading to a steep loss of clonal diversity over time. The transient expression of ZFNs caused an increase in clonal dynamics compared to lentiviral insertion as demonstrated by the following. First, there was a greater degree of clone loss (Figures 3 and 4; Additional file 8). Second, the top 10% of clones at PD 90 accounted for 89.2% of the population in the targeted library but only 74.8% of the population in the lentiviral cellular library. Finally, the percentage of clones that occupy the rare and abundant categories was higher in the targeted population at PD 90 (46% and 9%, respectively) compared to the lentiviral population (23% and 6%, respectively) (Additional file 3). It is possible that the ganciclovir treatment also contributed to the fall in clonal diversity but we found that populations of cells treated with ganciclovir alone did not have a perturbed spectrum of clonal representation compared to untreated cells, thus suggesting that the ganciclovir treatment had only a minimal impact on the clonal dynamics in the targeted insertion of barcodes by ZFNs (Additional file 9). In summary, the increased clonal dynamics induced by ZFN targeted integration and ganciclovir treatment was surprisingly greater than that induced by lentiviral insertion alone. This result is counter-intuitive as we expected that targeted integration of the barcode would have decreased clonal dynamics. It is well known that engineered nucleases create double-strand breaks at off-target sites leading to both insertions/deletions at the sites of these off-target breaks and perhaps to larger gross chromosomal rearrangements. This assay seems to be a sensitive measure of the functional toxicity of engineered nucleases and can perhaps serve as a novel functional assay for the potential safety of using engineered nucleases in gene therapy applications.

### Clonal dynamics of HeLa and HEK-293 T-cell lines

In order to determine whether our findings of persistent and ongoing clonal dynamics in K562 cells were representative of other cell types, we marked and tracked the clonal dynamics of both the HeLa and HEK-293 T-cell lines. We created both cellular barcode libraries from the same lentiviral prep used in the K562 cell experiments, and passaged them in an identical manner. The results show that while relatively few clones were lost over 90 population doublings, we did see some skewing of the distribution of clones over time as well as development of major clones (Additional files 10, 11, 12 and 13). As with the original K562 experiments, we saw only a small number of major clones that recurred in different biological replicates, and a number of major clones that were unique to a single population. These results indicate that the HeLa and HEK-293 T-cell lines, as with K562 cells, show significant clonal dynamics even under ideal culture conditions.

### The number of clones that contribute to 3T3 cell lines derived from mouse embryonic fibroblasts

The barcode system we describe here is applicable to a large number of biological questions, including quantifying the number and distribution of cells that contribute to downstream populations. To demonstrate this, we passaged barcode marked mouse embryonic fibroblasts in a 3T3 experiment [27] and found that a minimum of 0.7% of the fibroblasts transformed and contributed to the 3T3 population (data not shown).

### Using the barcode marking system to compare clonal dynamics *in vitro* versus xenografts

**Figure 5:**
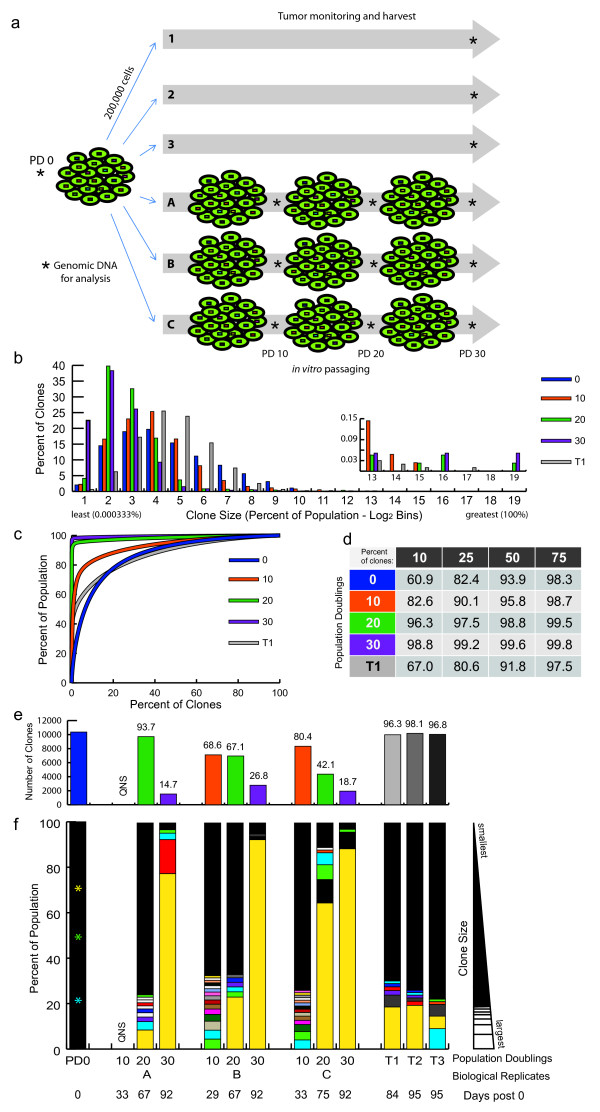
**HCC827 barcode libraries *in vitro *and *in vivo*. (a)** Diagram of HCC827 passaging experiments. **(b)** Clones were counted and binned in Log 2 bins based on percentage (frequency) within the population, from least to greatest. The percentage of clones in each bin is shown. Inset shows magnification of larger bins. HCC827 biological replicate A and tumor 1 are shown (others are shown in Additional files 14 (*in vitro*) and 15 (tumors)). **(c)** The percentage of clones, ranked from most to least frequent, plotted by what percentage of the population they made up. **(d)** The percentage of the population made up by the top indicated percentages of clones for each sample. **(e)** The number of clones found in each sample. **(f)** Rank order clones by percentage of the population for each sample; greatest to least. Any clones ≥1% are shown as white sections, the remaining population of clones smaller than 1% are represented by black in each column. The same barcode occurring as a major clone in more than one sample is marked by color. Stars in PD 0 column indicate positions of yellow, green, and blue clones in that sample. Days post-PD 0 are listed beneath each sample.

One of the important questions in cancer biology is the degree of selective pressure exerted by growing cells in culture (on plastic in 21% oxygen) versus growth *in vivo* as a mouse xenograft. We hypothesized that we could measure the selective pressures on clonal dynamics of tumor outgrowth *in vivo* and *in vitro*. We studied the tumorigenic non-small-cell lung cancer line HCC827 [28], and marked the cells with barcodes as previously described. Three biological replicates of cells were cultured on plastic, while the same number of cells were injected into the right flanks of three NU/NU mice and allowed to form tumors (Figure 5a). In the xenograft experiment, once the tumors stabilized in size (tumors 2 and 3) or the tumor volume reached 1 mm^3^ (tumor 1) the mice were sacrificed, and the tumors were harvested for barcode sequencing. In the *in vitro* experiment we analyzed the clonal representation of the population at PD 10, 20, and 30. Sequencing revealed that by PD 30, after 92 days in culture, each of the three independent biologic replicates in the *in vitro* populations became dominated by the same clone (Figure 5f, yellow). The results from the three tumors derived from the same clones injected into mice were surprising. While the dominant clone in the *in vitro* populations was still one of the major clones, the tumor populations had little clonal loss, thus maintaining a higher degree of polyclonality and greatly reduced clonal skewing compared to the *in vitro* populations (Figure 5b-f; Additional files 14 and 15), especially compared to PD 20 and 30 but even compared to PD 10 with respect to total number of clones. We determined the number of population doublings *in vitro* by simply counting the cells as they are being passaged. It is difficult to determine, however, the number of population doublings *in vivo* because a substantial, but unknown, fraction of transplanted cells would be expected to die during the initial transplantation and the rate of apoptosis *in vivo* is also unknown.

We hypothesized that insertional mutagenesis caused by the barcode integration may have played a role in the growth advantage seen in this clone. We mapped the barcode insertion site of this clone to the second intron of *MSRB3*, a methionine sulfide reductase on human chromosome 12. Karyotype analysis of the HCC827 cells used in these experiments show the presence of three copies of chromosome 12. We therefore believe it unlikely that the integration of the barcode in this clone is the causal factor in its distinct advantage over the other clones in the population because this gene has no reported role in tumor cell proliferation and would not disrupt the coding region of the gene.

### Quantifying changes in clonal representation using the Shannon-Weaver diversity index

The Shannon-Weaver diversity index is a powerful quantitative measure that accounts for both the number of different elements (in our case, cellular clones) and the relative representation of each element within the population (in our case, the relative abundance of each clone). It is broadly used in the ecology literature but applies very well to studies of clonal dynamics [29,30]. In the Shannon-Weaver diversity index, a higher number shows that the population is more diverse and evenly represented while a lower number demonstrates a more restricted and more unequal population. In all of our experiments, the Shannon-Weaver diversity index decreased, usually quite dramatically over time (Additional file 16).

## Discussion

We have developed a system that genetically marks individual cells, allowing for the simple, simultaneous, and quantitative tracking of thousands of cells using a combination of barcode marking and high-throughput sequencing. In establishing and validating this method we have focused on a system in which we can track >12,000 different clones simultaneously, but have also extended this to develop barcode libraries of varying complexities, including libraries that consist of over one million different barcodes (data not shown). Just as with the >12,000 complexity barcode library, we confirmed the complexity of these larger libraries by sequencing and they are now being used in other work to study the dynamics of hematopoietic stem cell reconstitution in non-human primates. With these larger libraries even greater care must be taken at each step (the creation of lentivirus, the marking of cells, and so on) to maintain the complexity and not create bottlenecks that would artificially skew the clonal barcode representation.

We have validated the power of this system by using it to track the spontaneous clonal dynamics of cell lines grown in culture and after xenotransplantation. We found that cell lines that have been grown in culture for years continue to have ongoing, dramatic clonal fluctuations, with many clones disappearing over time and others outgrowing their neighbors. When we started the analysis from a single cell, these effects were reduced but not abrogated, demonstrating that the process is ongoing and not static even within a group of closely related cells. These results quantify and confirm what is understood, but often underappreciated, namely that the 'same' cell line in different laboratories or at different times in the same laboratory may contain markedly different clonal repertoires and thus may display strikingly different behavior. We designed our studies to put the populations under as little stress as possible and yet we still observed dynamic ongoing population changes. It is likely that under situations in which populations are stressed, even more distinct clonal dynamics will be observed. Moreover, this system should allow the study of diverse clonal repertoires of populations resulting from ongoing clonal dynamics when cells are placed under different environmental pressures. One great use of this system would be to study the clonal dynamics of chemotherapeutic resistance when cancer cells are exposed to different agents (both classical and 'targeted' therapies). Our studies were not designed to investigate the mechanism of the ongoing dynamics but the rapid pace of change suggests that epigenetic alterations may be an important aspect of the process, in addition to the background genetic changes that occur in all cells over time.

In demonstrating the utility of this powerful clone tracking strategy in multiple systems, two of our findings contradict general consensus in their respective fields. The first major surprise was that the degree of clonal skewing (more clonal dropout, higher proportion of clones becoming dominant within the population) was greater when we used ZFNs to target the barcode to a single 'safe harbor' site in the genome, compared with using lentiviral vectors to semi-randomly integrate the barcode into uncontrolled sites in the genome. This finding suggests that other cellular characteristics, such as pre-determined genetic and epigenetic factors, must be considered in any gene editing strategy. This finding also highlights this strategy as a sensitive method to potentially interrogate factors that might affect both genetic and epigenetic stability. The second surprise was finding that there is less clonal selection pressure when passaging human cancer cells through a xenograft mouse model than when culturing in a plastic dish. This finding highlights how this method can be applied to monitor the effects of both cell intrinsic and cell extrinsic factors in clonal diversity in complex biological systems. Thus, barcode marking is a useful system to interrogate different methods for their ability to maintain the complexity of a population of cells over time. Moreover, the results support the continued use of mouse xenografting as a method to maintain the clonal complexity of human tumors and thus may represent a better model system for studying cancer than *in vitro* cell culture. For example, this system could be used to study the clonal dynamics of primary tumor development, local tumor recurrence following resection, metastasis and the clonal dynamics of chemotherapy resistance.

Finally, there are several other potential important future uses of our cellular barcoding system. One use is as a control for any small interfering RNA or small hairpin RNA screens to establish the background rate of false positives created by clonal heterogeneity and dynamics of the cell population being challenged. A second use would be to use the system to better understand the dynamics of cancer, including the frequency of tumor initiating/propagating cells in a population and the clonality of recurrence and metastasis. While the power of barcoding has already been shown by studying reconstitution of the hematopoietic system after transplantation, our system is designed for scale and enables the tracking of a larger number of cells. We can track the efficiency and clonality of hematopoietic engraftment in larger, more clinically relevant model systems over time. Moreover, the system described here can be used to evaluate different interventions that might affect either of these factors in larger model systems. Lastly, this barcoding system could also be used to study the clonal dynamics of somatic cell re-programming in induced pluripotent stem cells.

## Conclusions

We have developed a simple, cost-effective method to simultaneously track the fate of tens of thousands of cells using a DNA molecular barcode marking system. We believe this new method will be of broad use in a range of scientific fields, some of which we have outlined here, and is likely to lend insights into clonal population dynamics that are not possible using other available methods.

## Materials and methods

### Library construction

The Illumina P5 adapter sequence was cloned upstream of UBC-GFP in pLGR7, a third generation lentiviral vector with self-inactivating long terminal repeats. Barcode oligos were synthesized by Integrated DNA Technologies (Coralville, IA, USA) and contained 20 randomized bases flanked by known anchor sequences and enzyme restriction sites. Approximately 10^12^ barcode oligos were annealed to a short (20 bp) oligo complementary to the 3’ end, followed by extension by Phusion polymerase to create double-stranded barcode fragments. These fragments were then cloned immediately downstream of the P5 adapter sequence within the vector. Pooled ligations were electroporated into *Escherichia coli* and expanded in SOC medium for 16 hours at 37°C with shaking. We plated 0.05% of this transformation on an antibiotic resistance plate and colonies were counted after overnight growth. The 1,063 colonies suggest the complexity of the remaining library to be approximately 20,000. Plasmid libraries were harvested and purified with an EndoFree Plasmid Maxi Kit (QIAGEN, Valencia, CA, USA).

### Cell lines and cell culture

K562 and HCC827 cells were obtained from ATCC (Manassas, VA, USA) and maintained in RPMI 1640 (Hyclone, Logan, UT, USA) supplemented with 10% Bovine Growth Serum (Hyclone, Logan, UT, USA), 2 mM L-gluatmine, 100 units/ml penicillin, and 100 μg/ml streptomycin (Mediatech, Manassas, VA, USA). HEK-293T cells were obtained from ATCC and HeLa cells were a generous gift from Dr Alejandro Sweet-Cordero (Stanford University). Both were maintained in Dulbeco’s modified Eagle’s medium (Hyclone) supplemented with 10% Bovine Growth Serum (Hyclone), 2 mM L-gluatmine, 100 units/ml penicillin, and 100 μg/ml streptomycin (Mediatech).

### Lentivirus production

Barcode lentivirus was produced in HEK-293 T cells by calcium phosphate transfection of 10 μg barcode plasmid vector along with 3 μg VSVG, 5 μg RRE, and 2.5 μg RSV/REV helper plasmids per 10 cm plate. At 18 hours post-transfection, fresh media with 4 mM caffeine was added to cells [31]. Virus-containing supernatant was collected 24 hours later and concentrated by ultracentrifugation, aliquoted, and stored at -80°C.

### Cellular barcode libraries and passaging experiments

For lentiviral barcoding, 2 × 10^6^ cells were infected at low MOIs such that 5 to 10% of cells expressed GFP, in order to reduce the chances of any single cell being marked by more than one barcode. GFP-expressing cells were sorted from each population 4 days after transduction. These cells were expanded in culture for an additional 4 to 5 days, resulting in the PD 0 population. Aliquots of 3 × 10^5^ PD 0 cells were used to start the parallel biological replicate populations A, B, and C for each cell line or condition. Additional PD 0 cells were harvested for genomic DNA for barcode analysis, and remaining cells were frozen and stored for future use.

Cells were passaged every 3 days by trypsinization if necessary for attached cells, mixing well and taking 3 × 10^5^ live cells to a new well with fresh medium. Cells were monitored by flow cytometry for GFP expression as well as cell counts. At PD 30, 60, and 90, cells were harvested for genomic DNA as outlined below.

### Targeted barcode insertion

ZFNs designed to target the human *CCR5* locus have been described previously [24]. The barcode targeting vector was created by cloning barcode oligos and the P5 Illumina adapter, similar to the lentiviral barcode library, downstream of the UBC-GFP within the homology arms of a CCR5 targeting vector described previously [24]. Included outside the homology arms was an HSV-TK domain for negative selection.

K562 cells were nucleofected (Lonza, Basel, Switzerland) with 10 μg targeting vector and 1 μg of each ZFN plasmid using program T-016 and nucleofection buffer containing 100 mM KH_2_PO_4_, 15 mM NaHCO_3_, 12 mM MgCl_2_•6 H_2_0, 8 mM adenosine 5′-triphosphate, 2 mM glucose, pH 7.4. Negative selection was performed with two pulses of 5 μM ganciclovir at days 5 and 11 post-transfection.

### Xenograft experiments

Eight-week-old female NU/NU athymic mice were obtained from Charles River Laboratories (Wilmington, MA, USA). 2 × 10^5^ barcode-marked HCC827 cells in 200 μl phosphate-buffered saline were injected sub-cutaneously into the right flank of each mouse. Mice were maintained at Stanford University’s Research Animal Facility and monitored weekly until tumors were palpable and twice weekly thereafter. Animal procedures were approved by the International Animal Care and Use Committee.

### DNA barcode extraction and sequencing

Genomic DNA from both cultured cells and tumors was harvested with the DNeasy Blood and Tissue Kit (QIAGEN). Barcodes were amplified by PCR using Phusion high-fidelity DNA polymerase (New England Biolabs, Beverly, MA, USA) with primers containing the adapters necessary for Illumina sequencing. Forward primers also contained 4 bp indexing tags to allow sample multiplexing. PCR bands were size-selected on 1.1% agarose gel and purified with a QIAquick Gel Extraction Kit (QIAGEN). DNA concentrations were checked by BioAnalyzer and high-throughput sequencing was performed using the Illumina GA II sequencer by the Stanford Functional Genomics Facility.

### Barcode sequencing data analysis

We developed a computational tool to enable rapid readout of barcodes from raw Illumina FASTQ data. All methods and documentation are available via Github at [18]. The software is open source and can be modified as needed to suit additional applications. The Illumina FASTQ data from these runs are available in the NIH Sequence Read Archive as project [SRA:SRP029299].

### Statistics

The Shannon-Weaver diversity index for each sample was calculated by the formula:

$$\begin{array}{ll} \;H=-\Sigma & p_{i}\ln\left(p_{i}\right)\;\text{where}\;p_{i}\;\mathrm{is}\;\text{the}\;\text{frequency}\;\mathrm{of}\;\text{barcode}\;i\;\mathrm{in}\\ & a\;\text{given}\;\text{sample}. \end{array}$$

Monte Carlo simulation of the cell culture passaging experiments was performed and showed that, for experiments in which 3 × 10^5^ cells were maintained at each passage, approximately 80 to 84% of clones should be present at PD 90 if the growth rate of all clones is equal. For experiments in which 2 × 10^6^ cells were passaged, 100% of clones would be expected to persist at PD 90. Additional details of the simulations are available via Github at [18], including the histogram plots of various simulations. Briefly, we started the simulation with 300,000 cells barcoded with one of 14,000 unique identifiers. The cells were assumed to be evenly divided between the barcode identifiers.

After this, we perform the following steps repeatedly. First, the cell population is grown. The number of cells of each population is increased to reflect exponential growth of the population. We use a rate consistent with a doubling every 19 hours (this rate was determined experimentally by measuring the population doubling time of our K562 cells); this yields approximately 13-fold growth across 3 days (1 passage). Second, after 3 days, we randomly select 300,000 cells from the population (should be around 4,000,000 after 3 days of growth). Third, we report the number of unique barcodes present in the 300,000 selected cells.

The above is simulated for 25 passages (or 75 days or approximately 94 population doublings).

## Abbreviations

bp: base pair; eGFP: enhanced green fluorescent protein; MOI: multiplicity of infection; PCR: polymerase chain reaction; PD: population doubling; UBC: ubiquitin-C; ZFN: zinc-finger nuclease.

## Competing interests

The authors declare that they have no competing interests.

## Authors’ contributions

SNP designed and performed experiments, analyzed the data and wrote the manuscript. LCB analyzed data. DM helped design experiments, analyzed the data and wrote the manuscript. MP designed experiments, analyzed the data and wrote the manuscript. All authors read and approved the final manuscript.

## Supplementary Material

Additional file 1 Pairwise comparisons of barcode frequency for each of the plasmid library sequencing replicates. Click here for file

Additional file 2 K562 biological replicates B and C. Click here for file

Additional file 3 Percentage rare and abundant clones in each experimental sample. Click here for file

Additional file 4 Tracking major K562 clones in all populations. Click here for file

Additional file 5 K562 cells derived from a single cell. Click here for file

Additional file 6 K562 cells derived from a single cell biological replicates B and C. Click here for file

Additional file 7 Targeted K562 biological replicates B and C. Click here for file

Additional file 8 Area-proportional Venn diagrams comparing clone overlap among biological replicates over time across K562 experiments. Click here for file

Additional file 9 Effects of etoposide or ganciclovir on clonality. Click here for file

Additional file 10 HEK-293 T barcode passaging experimental results. Click here for file

Additional file 11 HEK-293 T biological replicates B and C. Click here for file

Additional file 12 HeLa barcode passaging experimental results. Click here for file

Additional file 13 HeLa biological replicates B and C. Click here for file

Additional file 14 **HCC827 *in vitro *** **biological replicates B and C.** Click here for file

Additional file 15 HCC827 xenograft tumors. Click here for file

Additional file 16 Shannon-Weaver diversity indices over time for each sample. Click here for file
