# Supplementary material for: Lentiviral and targeted cellular barcoding reveals ongoing clonal dynamics of cell lines in vitro and in vivo
Source: Genome Biol. 2014 May 30;15(5):R75. doi: 10.1186/gb-2014-15-5-r75 (PMC4073073; doi:10.1186/gb-2014-15-5-r75)
Supplement: Additional file 1 — Pairwise comparisons of barcode frequency for each of the plasmid library sequencing replicates. [file gb-2014-15-5-r75-S1.pptx]

## Slide 1
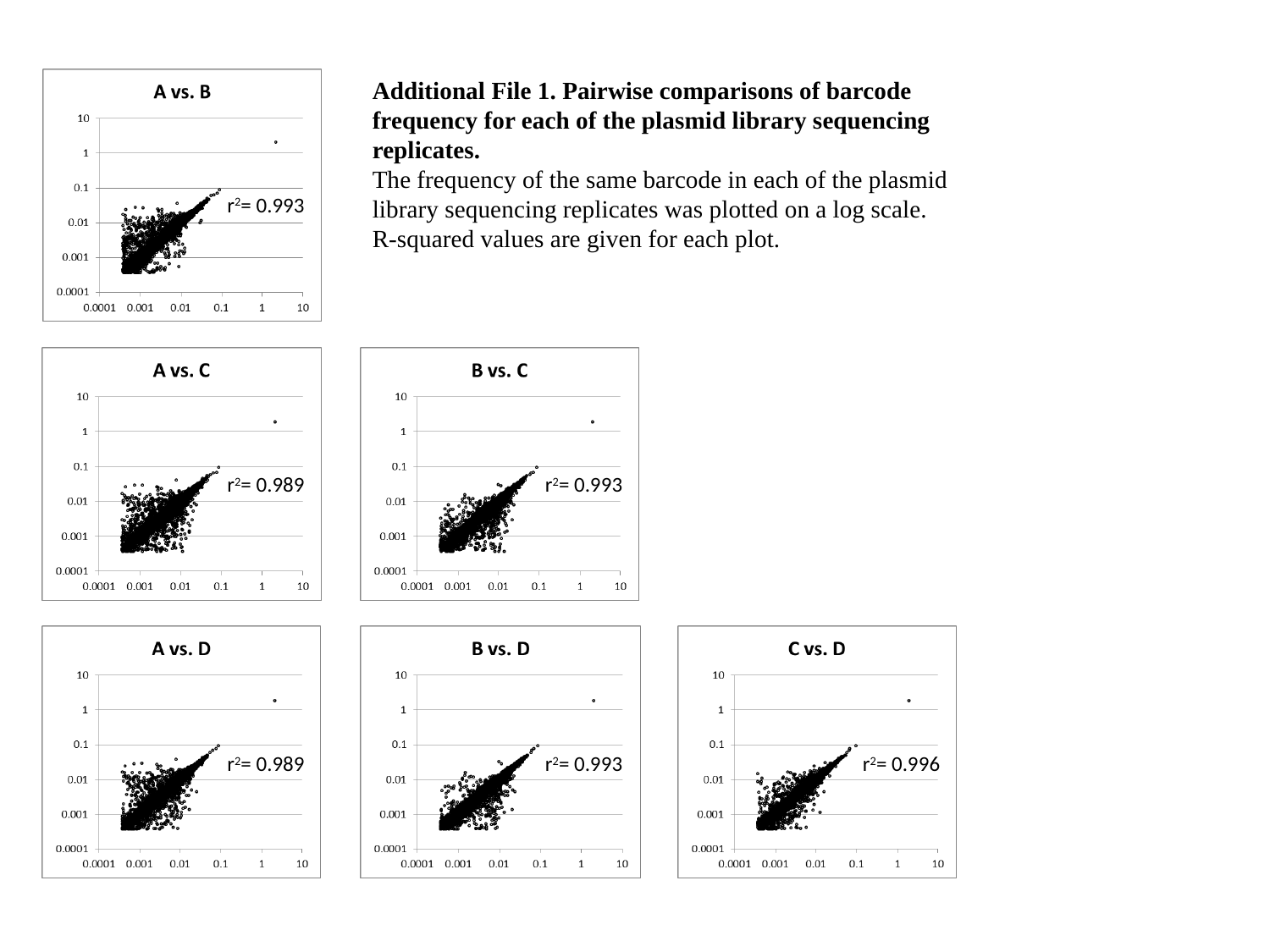

Additional File 1. Pairwise comparisons of barcode frequency for each of the plasmid library sequencing replicates.
The frequency of the same barcode in each of the plasmid library sequencing replicates was plotted on a log scale. R-squared values are given for each plot.
r2= 0.993
r2= 0.989
r2= 0.993
r2= 0.989
r2= 0.993
r2= 0.996
