# Supplementary material for: Lentiviral and targeted cellular barcoding reveals ongoing clonal dynamics of cell lines in vitro and in vivo
Source: Genome Biol. 2014 May 30;15(5):R75. doi: 10.1186/gb-2014-15-5-r75 (PMC4073073; doi:10.1186/gb-2014-15-5-r75)
Supplement: Additional file 2 — K562 biological replicates B and C. [file gb-2014-15-5-r75-S2.pptx]

## Slide 1
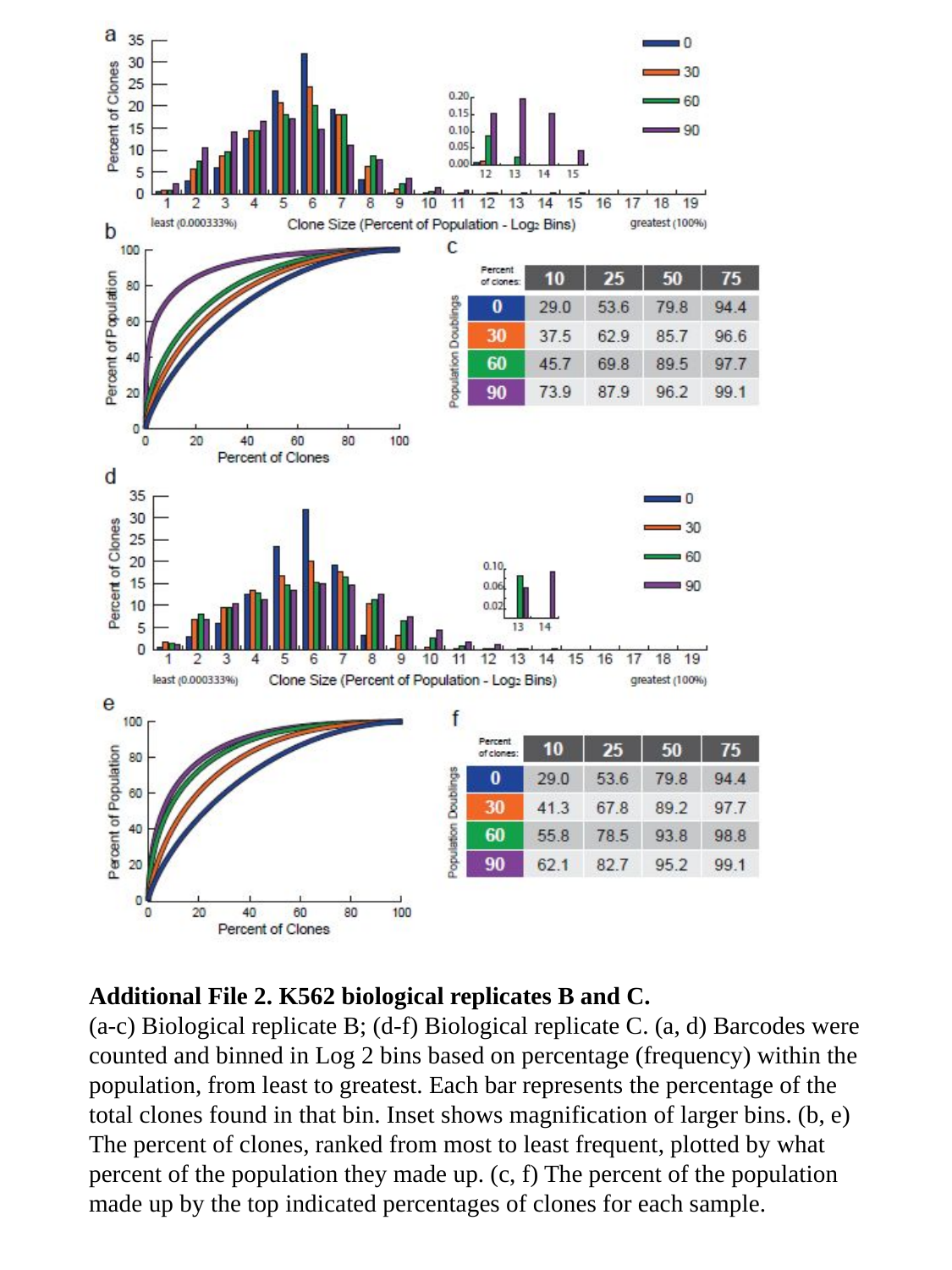

Additional File 2. K562 biological replicates B and C.
(a-c) Biological replicate B; (d-f) Biological replicate C. (a, d) Barcodes were counted and binned in Log 2 bins based on percentage (frequency) within the population, from least to greatest. Each bar represents the percentage of the total clones found in that bin. Inset shows magnification of larger bins. (b, e) The percent of clones, ranked from most to least frequent, plotted by what percent of the population they made up. (c, f) The percent of the population made up by the top indicated percentages of clones for each sample.
