# Supplementary material for: Lentiviral and targeted cellular barcoding reveals ongoing clonal dynamics of cell lines in vitro and in vivo
Source: Genome Biol. 2014 May 30;15(5):R75. doi: 10.1186/gb-2014-15-5-r75 (PMC4073073; doi:10.1186/gb-2014-15-5-r75)
Supplement: Additional file 3 — Percentage rare and abundant clones in each experimental sample. [file gb-2014-15-5-r75-S3.pptx]

## Slide 1
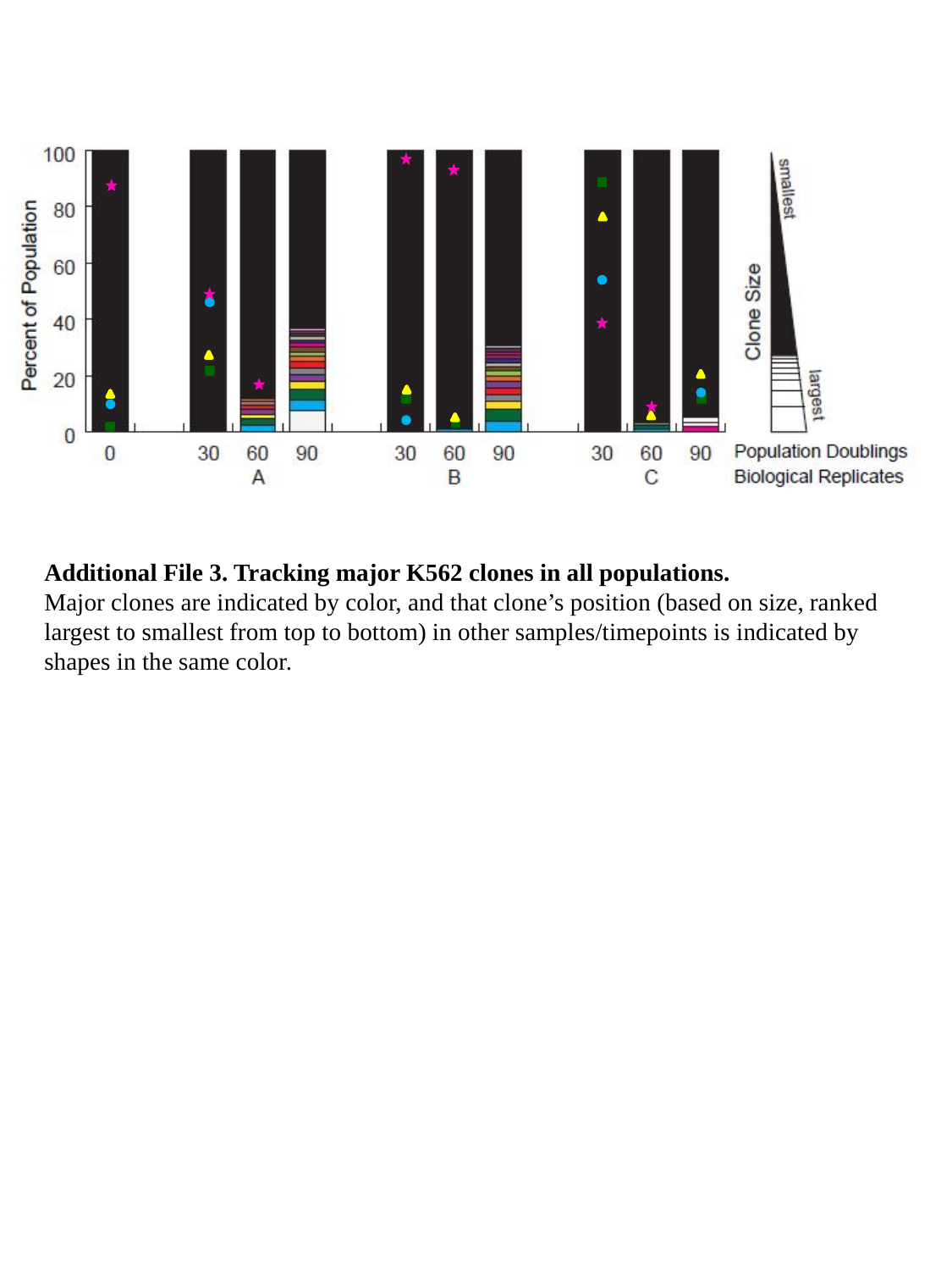

Additional File 3. Tracking major K562 clones in all populations.
Major clones are indicated by color, and that clone’s position (based on size, ranked largest to smallest from top to bottom) in other samples/timepoints is indicated by shapes in the same color.
