# Supplementary material for: Lentiviral and targeted cellular barcoding reveals ongoing clonal dynamics of cell lines in vitro and in vivo
Source: Genome Biol. 2014 May 30;15(5):R75. doi: 10.1186/gb-2014-15-5-r75 (PMC4073073; doi:10.1186/gb-2014-15-5-r75)
Supplement: Additional file 4 — Tracking major K562 clones in all populations. [file gb-2014-15-5-r75-S4.pptx]

## Slide 1
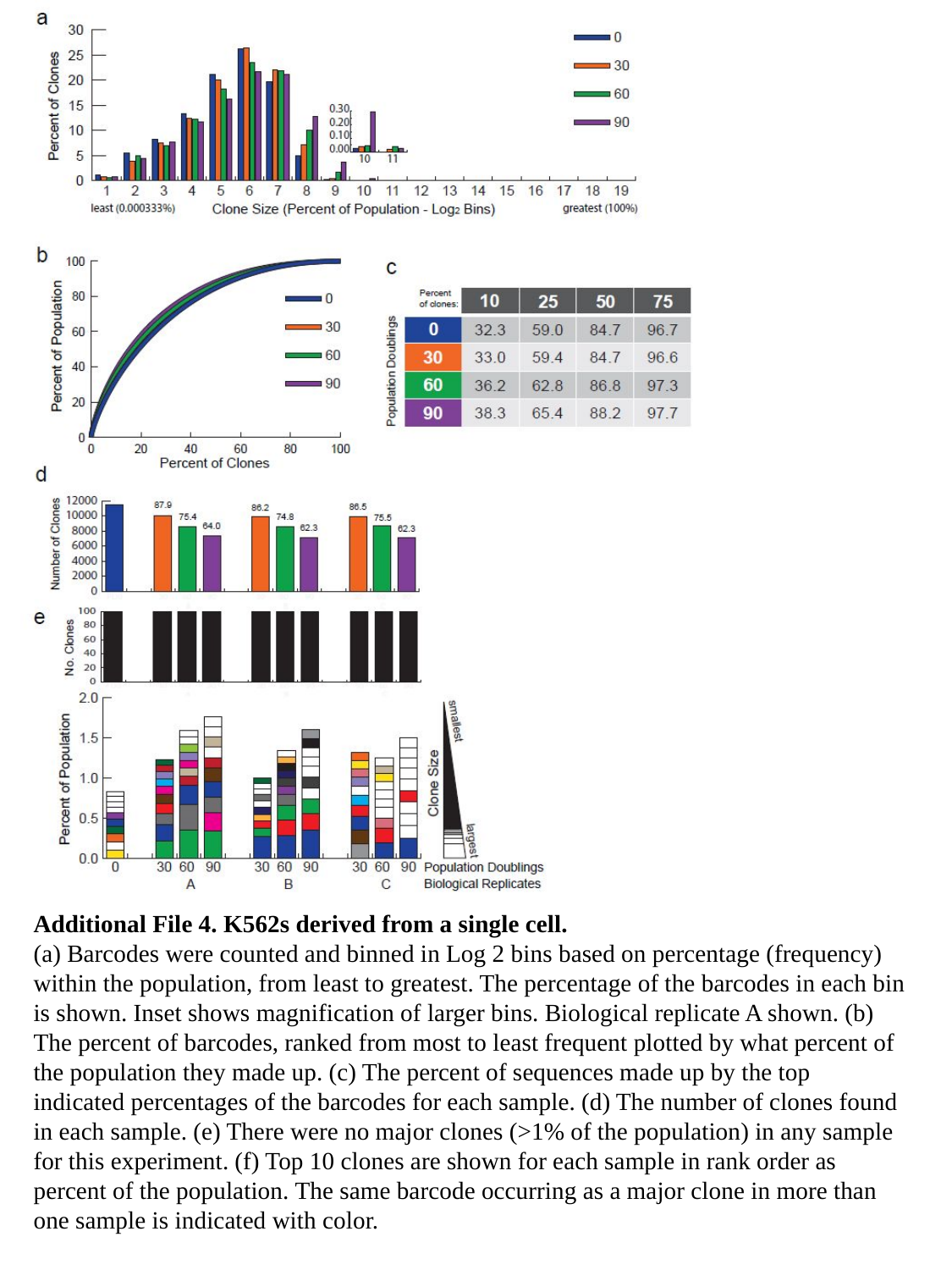

Additional File 4. K562s derived from a single cell.
(a) Barcodes were counted and binned in Log 2 bins based on percentage (frequency) within the population, from least to greatest. The percentage of the barcodes in each bin is shown. Inset shows magnification of larger bins. Biological replicate A shown. (b) The percent of barcodes, ranked from most to least frequent plotted by what percent of the population they made up. (c) The percent of sequences made up by the top indicated percentages of the barcodes for each sample. (d) The number of clones found in each sample. (e) There were no major clones (>1% of the population) in any sample for this experiment. (f) Top 10 clones are shown for each sample in rank order as percent of the population. The same barcode occurring as a major clone in more than one sample is indicated with color.
