# Supplementary material for: Lentiviral and targeted cellular barcoding reveals ongoing clonal dynamics of cell lines in vitro and in vivo
Source: Genome Biol. 2014 May 30;15(5):R75. doi: 10.1186/gb-2014-15-5-r75 (PMC4073073; doi:10.1186/gb-2014-15-5-r75)
Supplement: Additional file 5 — K562 cells derived from a single cell. [file gb-2014-15-5-r75-S5.pptx]

## Slide 1
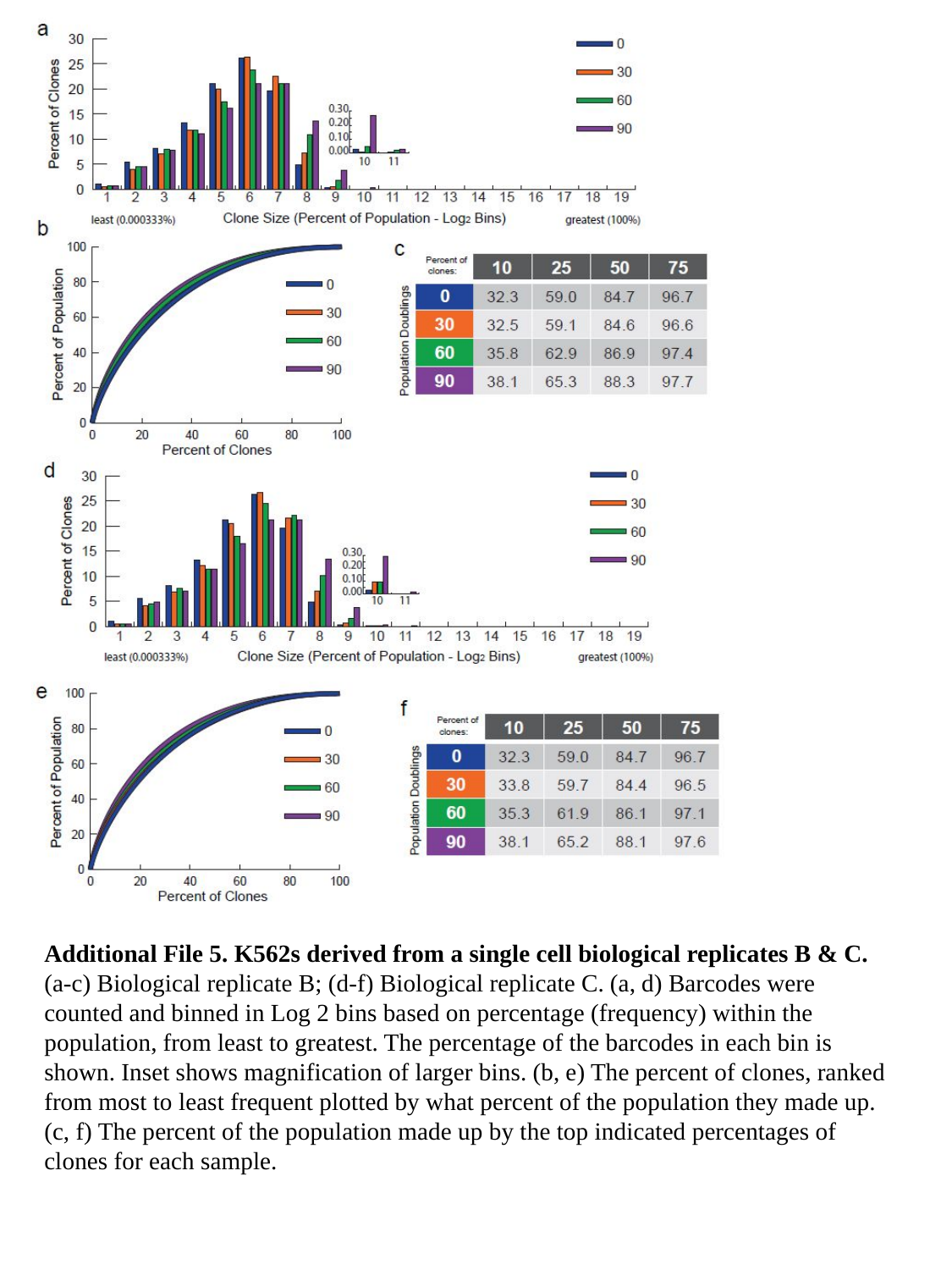

Additional File 5. K562s derived from a single cell biological replicates B & C.
(a-c) Biological replicate B; (d-f) Biological replicate C. (a, d) Barcodes were counted and binned in Log 2 bins based on percentage (frequency) within the population, from least to greatest. The percentage of the barcodes in each bin is shown. Inset shows magnification of larger bins. (b, e) The percent of clones, ranked from most to least frequent plotted by what percent of the population they made up. (c, f) The percent of the population made up by the top indicated percentages of clones for each sample.
