# Supplementary material for: Lentiviral and targeted cellular barcoding reveals ongoing clonal dynamics of cell lines in vitro and in vivo
Source: Genome Biol. 2014 May 30;15(5):R75. doi: 10.1186/gb-2014-15-5-r75 (PMC4073073; doi:10.1186/gb-2014-15-5-r75)
Supplement: Additional file 6 — K562 cells derived from a single cell biological replicates B and C. [file gb-2014-15-5-r75-S6.pdf]

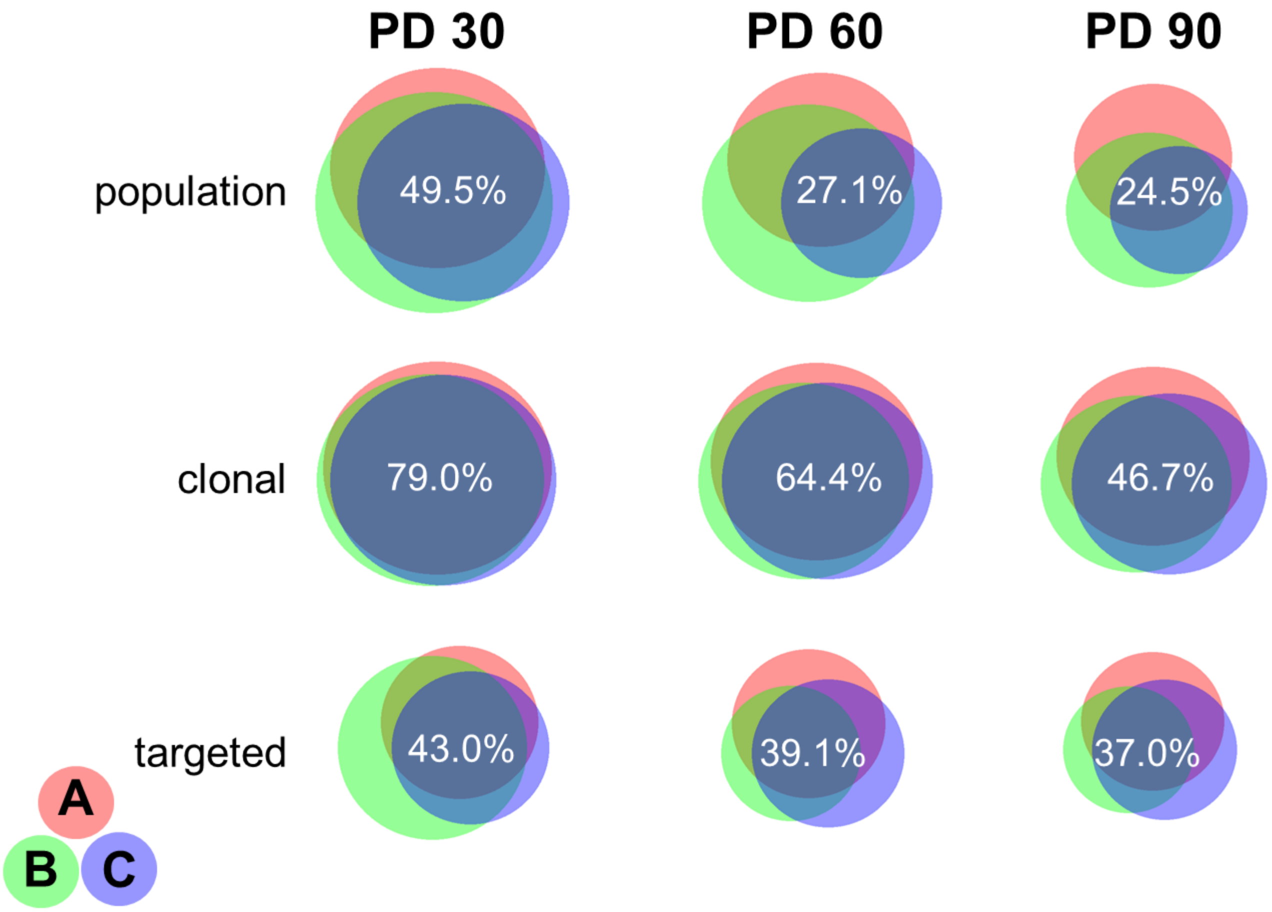

Additional File 6. Area-proportional Venn diagrams comparing clone overlap among biological replicates at given time points across K562 experiments.
The proportions of each sample that overlap with each of the other two biological replicates, indicating the relative similarity of each replicate to the others. The percent of clones in all three biological replicates is listed.
BioVenn web application available at http://www.cmbi.ru.nl/cdd/biovenn/
