# Supplementary material for: Lentiviral and targeted cellular barcoding reveals ongoing clonal dynamics of cell lines in vitro and in vivo
Source: Genome Biol. 2014 May 30;15(5):R75. doi: 10.1186/gb-2014-15-5-r75 (PMC4073073; doi:10.1186/gb-2014-15-5-r75)
Supplement: Additional file 7 — Targeted K562 biological replicates B and C. [file gb-2014-15-5-r75-S7.pdf]

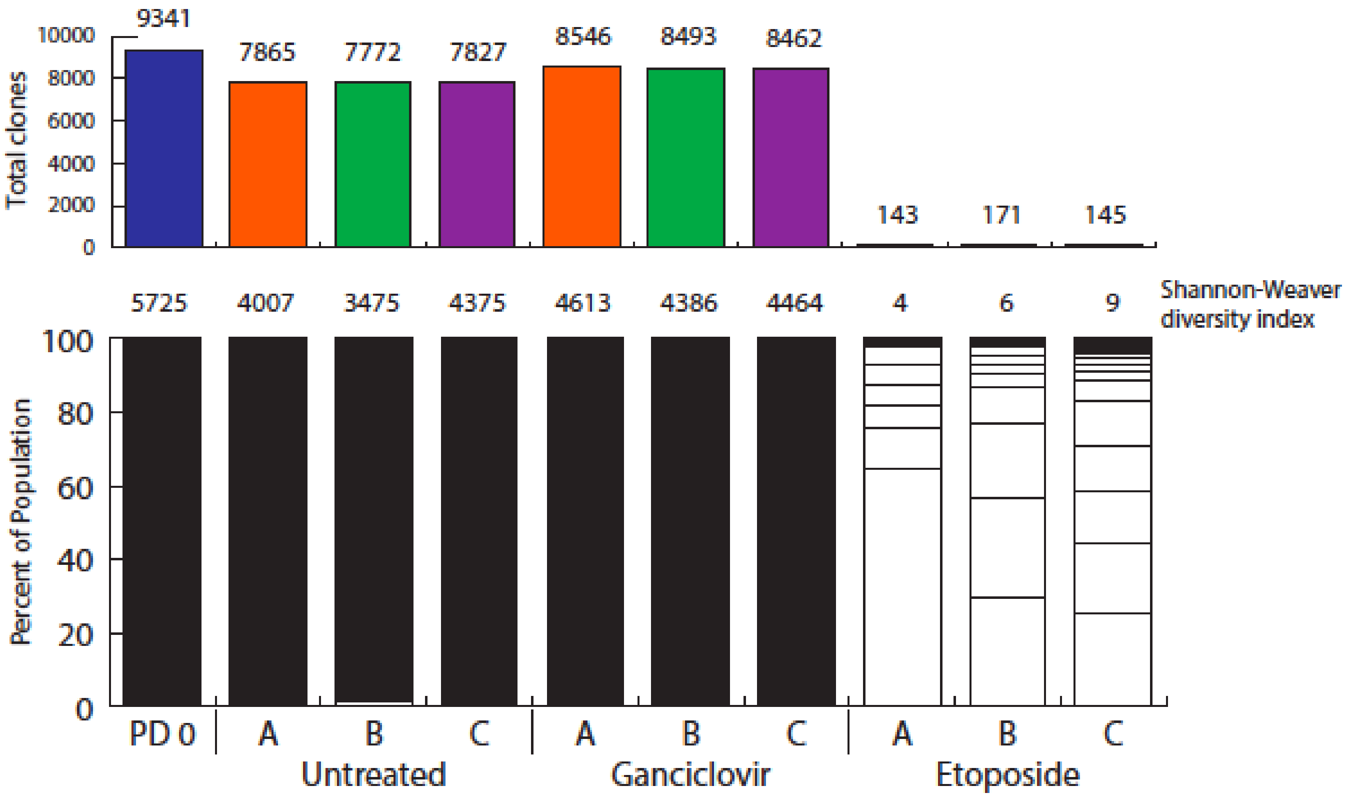

Additional File 7. Effects of etoposide or ganciclovir on clonality.
One million barcoded K562 cells were treated with 5uM ganciclovir (for three days) or 22nM etoposide (for 18 hours) or were left untreated, performed in triplicate (A-C). Populations were passaged for 30 population doublings (PD) before barcode sequencing. The number of clones found in each sample is indicated above each column (top). Bottom row of data indicate all clones in rank order by percent of the population. Any clones ≥1% are delimited by white sections, the remaining population of clones smaller than 1% are represented by black in each column. There were no shared major clones among the samples.Shannon-Weaver diversity index values are indicated for each sample. Exposure to etoposide had a profound effect on the clonal makeup of those populations, while ganciclovir treatment did not appear to alter cell growth or dynamics.
