# Supplementary material for: Lentiviral and targeted cellular barcoding reveals ongoing clonal dynamics of cell lines in vitro and in vivo
Source: Genome Biol. 2014 May 30;15(5):R75. doi: 10.1186/gb-2014-15-5-r75 (PMC4073073; doi:10.1186/gb-2014-15-5-r75)
Supplement: Additional file 9 — Effects of etoposide or ganciclovir on clonality. [file gb-2014-15-5-r75-S9.pdf]

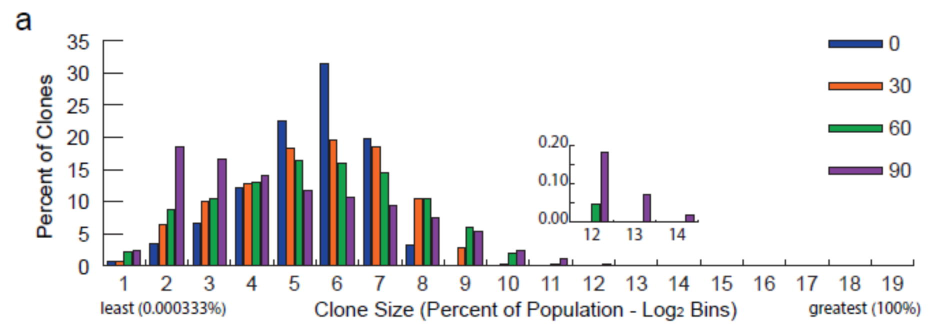

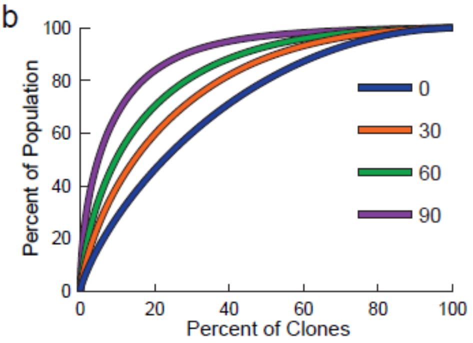

c

Population Doublings / Percent of clones:	10	25	50	75
0	28.4	53.5	80.2	94.8
30	40.0	66.8	88.6	97.4
60	51.2	76.5	92.9	98.6
90	67.6	88.1	97.0	99.2

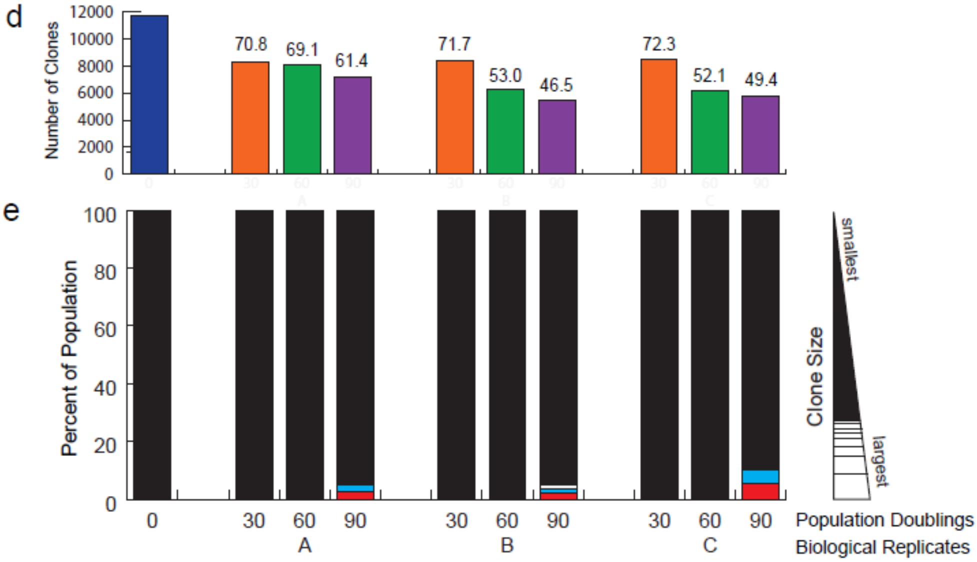

Additional File 9. HEK-293T barcode passaging experimental results.
(a) Barcodes were counted and binned in Log 2 bins based on percentage (frequency) within the population, from least to greatest. The percentage of the barcodes in each bin is shown. Inset shows magnification of larger bins. Biological replicate A shown. (b) The percent of barcodes, ranked from most to least frequent plotted by what percent of the population they made up. (c) The percent of sequences made up by the top indicated percentages of the barcodes for each sample. (d) The number of clones found in each sample. (e) All clones in rank order by percent of the population. Any clones ≥1% are delimited by white sections, the remaining population of clones smaller than 1% are represented by black in each column. The same barcode occurring as a major clone in more than one sample is indicated with color.
