# Supplementary material for: Lentiviral and targeted cellular barcoding reveals ongoing clonal dynamics of cell lines in vitro and in vivo
Source: Genome Biol. 2014 May 30;15(5):R75. doi: 10.1186/gb-2014-15-5-r75 (PMC4073073; doi:10.1186/gb-2014-15-5-r75)
Supplement: Additional file 13 — HeLa biological replicates B and C. [file gb-2014-15-5-r75-S13.pdf]

		PD 0	PD 30	PD 60	PD 90
**K562**	*rare*	4.65	9.82	15.75	23.27
	abundant	0.17	2.22	5.33	6.01
**clonal K562**	*rare*	9.99	8.28	9.47	9.04
	abundant	0.27	0.43	1.56	3.45
**targeted K562**	*rare*	28.43	34.78	35.46	46.07
	abundant	2.80	7.15	12.36	9.07
**HeLa**	*rare*	5.37	13.11	20.62	34.46
	abundant	0.10	1.37	1.90	3.48
**HEK-293T**	*rare*	6.78	9.32	19.20	31.10
	abundant	0.12	2.96	7.25	7.76

		PD 0	PD 10	PD 20	PD 30	T1	T2	T3
**HCC827**	*rare*	23.87	32.08	53.81	74.37	17.55	14.10	13.37
	abundant	3.91	2.31	0.62	0.39	1.05	0.83	0.90

Additional File 13. Percent rare and abundant clones in each experimental sample.

The table shows the percent of clones classified as “rare” (<0.0007 % of the population) or “abundant” (>0.5% of the population) in each sample. This demonstrates the general trend of the distribution of clones moving toward both extremes over time.
