# Supplementary material for: Lentiviral and targeted cellular barcoding reveals ongoing clonal dynamics of cell lines in vitro and in vivo
Source: Genome Biol. 2014 May 30;15(5):R75. doi: 10.1186/gb-2014-15-5-r75 (PMC4073073; doi:10.1186/gb-2014-15-5-r75)
Supplement: Additional file 16 — Shannon-Weaver diversity indices over time for each sample. [file gb-2014-15-5-r75-S16.pdf]

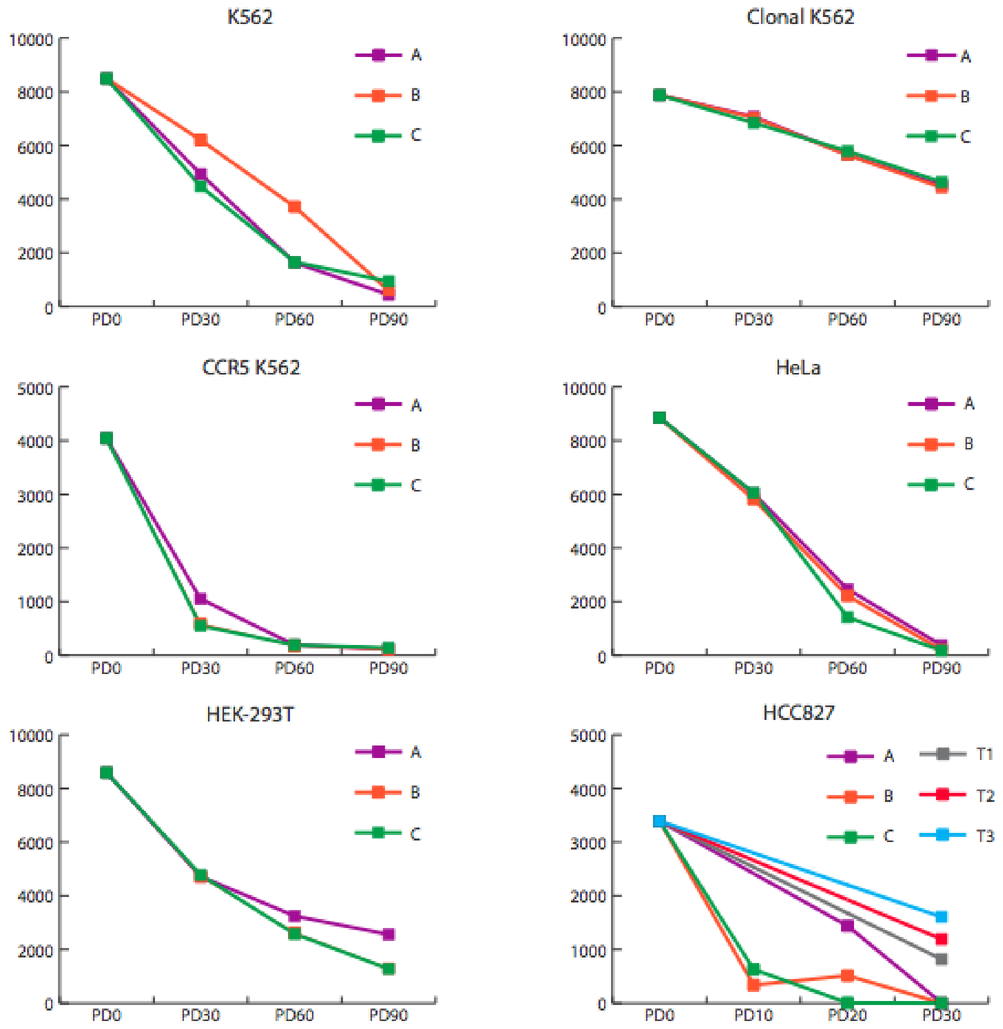

Additional File 16. Shannon-Weaver diversity indices.
Diversity index values are plotted over time for each biological replicate from each experiment.
